# Adult life outcomes for adolescents 12.4 years after dialectical behavior therapy - a randomized clinical trial

**DOI:** 10.1007/s00787-025-02856-w

**Published:** 2026-01-16

**Authors:** Lars Mehlum, Iselin Solerød Dibaj, Egil Haga, Sofie Egidius Helle, Katharina T. E. Morken, Ole Klungsøyr, Anita J. Tørmoen

**Affiliations:** 1 National Centre for Suicide Research and Prevention, Institute of Clinical Medicine, University of Oslo, Oslo, Norway; 2 Department of Addiction Medicine, Haukeland University Hospital, Bergen, Norway; 3 Department of Clinical Psychology, University of Bergen, Bergen, Norway

**Keywords:** Self-harm, Attempted suicide, Psychotherapy, Longitudinal

## Abstract

Adolescents with suicidal and self-harming behavior run significant risk for suicide. Outcomes in adult life after specialized treatment for this population are unknown. We examined adult life outcomes in adolescents with recurrent suicidal and self-harming behavior and borderline features having participated in a randomized trial of dialectical behavior therapy (DBT-A) or enhanced usual care (EUC). Of the original 77 study participants, 61 (79%) were followed-up at a mean age of 28 years, an average of 12.4 years post-baseline. Primary outcomes were frequency of self-harm episodes and levels of suicidal ideation and depressive symptoms over the follow-up intervals. Secondary outcomes were borderline symptoms, psychiatric morbidity, occupational and psychosocial functioning. Self-harm frequency was consistently lower throughout the 12.4 year follow-up period in participants who had received DBT-A, although differences were not statistically significant at the final follow-up. Whereas suicide attempts were infrequent in both groups, self-harm episodes were more than twice as common in the EUC group compared to the DBT-A group with a mean difference of 90 episodes per person from baseline to 12.4 years follow-up. DBT-A was associated with significantly stronger reductions in suicidal ideation, depressive and borderline symptoms at end of treatment but differences tapered off until not statistically significant at the final follow-up. There were no significant trend differences in current psychiatric morbidity, self-reported depressive symptoms and global functioning. This first study of adult life outcomes of specialized psychosocial interventions for adolescents with recurrent suicidal and self-harming behavior and borderline features suggests that adolescents may have a realistic hope of sustained recovery as adults.

Trial Registration: http://ClinicalTrials.gov/;NCT04298190

## Introduction

Suicide remains a leading cause of death in youths world-wide [1] and a major public health concern; in the US suicide remains a top priority research area for the NIMH [2]. More frequently occurring, non-fatal self-harm (self-poisoning or self-injury with or without suicide intent) [3] has been strongly increasing in prevalence among youths in many countries [4–6]. Non-fatal self-harm is a strong predictor of self-harm repetition [7, 8] and of future suicide [9] particularly among adolescents where a more than 30-fold increase in the one-year incidence of suicide has been found among those who have self-harmed compared to the expected rate in the general population [10]. Self-harm has many known risk factors [11], among them emotion dysregulation [12] which peaks in adolescence [13] and which is associated with reduced quality of life and functional incapacity in adulthood [14]. Several psychosocial interventions targeting adolescents presenting with suicidal and self-harming behaviors have been developed and put to trial for their efficacy over the last 1–2 decades [15], but to date, only for Dialectical Behavior Therapy (DBT) research is sufficient to label it an evidence based treatment [16] justifying inclusion in clinical guidelines [17]. We have previously demonstrated that a 19 weeks version of DBT adapted for adolescents (DBT-A)[18] effectively reduced frequency of self-harm episodes, suicidal ideation and depressive symptoms more than enhanced usual care (EUC) of about the same duration and intensity [19]. The first randomized trial to demonstrate the effectiveness of DBT-A, its findings have later been replicated in several trials [20, 21]. Our finding that DBT-A significantly reduced self-harm post-treatment was maintained at 1.6 and 3.1 year post-baseline follow-ups [22, 23]. However, how adolescents who have received psychosocial interventions fare as they enter adulthood is largely unknown due to a general lack of studies with sufficient follow-up time, prospective and controlled design, and adequate participation rates. Whether specialized treatment targeting recurrent self-harm and suicidal behaviors will lead to improved outcomes in adulthood is also unknown due to an absence of relevant studies.

Recurrent suicidal and self-harming behaviors in adolescents are strongly associated with problems of regulating emotions frequently linked to borderline personality disorder (BPD), either as a fully developed clinical disorder or some features of it [24]. BPD has a prevalence of 1–2% in the general population of adolescents up to 16 years old, but much higher in clinical populations [25], particularly among self-harming adolescents [24]. BPD and associated symptoms in adolescents are available to treatment [26, 27], but how BPD related symptomatology develops in adolescents after treatment as they enter adult life is sparingly investigated. The only study known to us is Jorgensen et al.'s recently published [28] five-year single cohort follow-up after treatment of adolescents with BPD, then in their late teens or early twenties, showing generally poor outcomes. Among non-clinical cohort studies of individuals with adolescent-onset BPD, Bornovalova et al.'s 10-year follow-up [29] is notable. As suggested by this study, and by several 10–20 year follow-up studies in adults with BPD, outcomes may improve considerably [30] over longer time perspectives. Such long-term follow-up studies of adolescents with BPD or BPD-features after specialized treatment are, however, lacking.

In the present study our aims were, thus, to investigate the long-term course and outcome in adolescents with recent and recurrent self-harm behaviors and BPD features who had received either DBT-A or EUC as they entered adulthood 12.4 years^1^ later with respect to frequency of suicide attempts and episodes of non-suicidal self-injury, severity of suicidal ideation, depressive and borderline symptoms, psychiatric morbidity, occupational and psychosocial functioning.

## Methods

### Participants

**Fig. 1:**
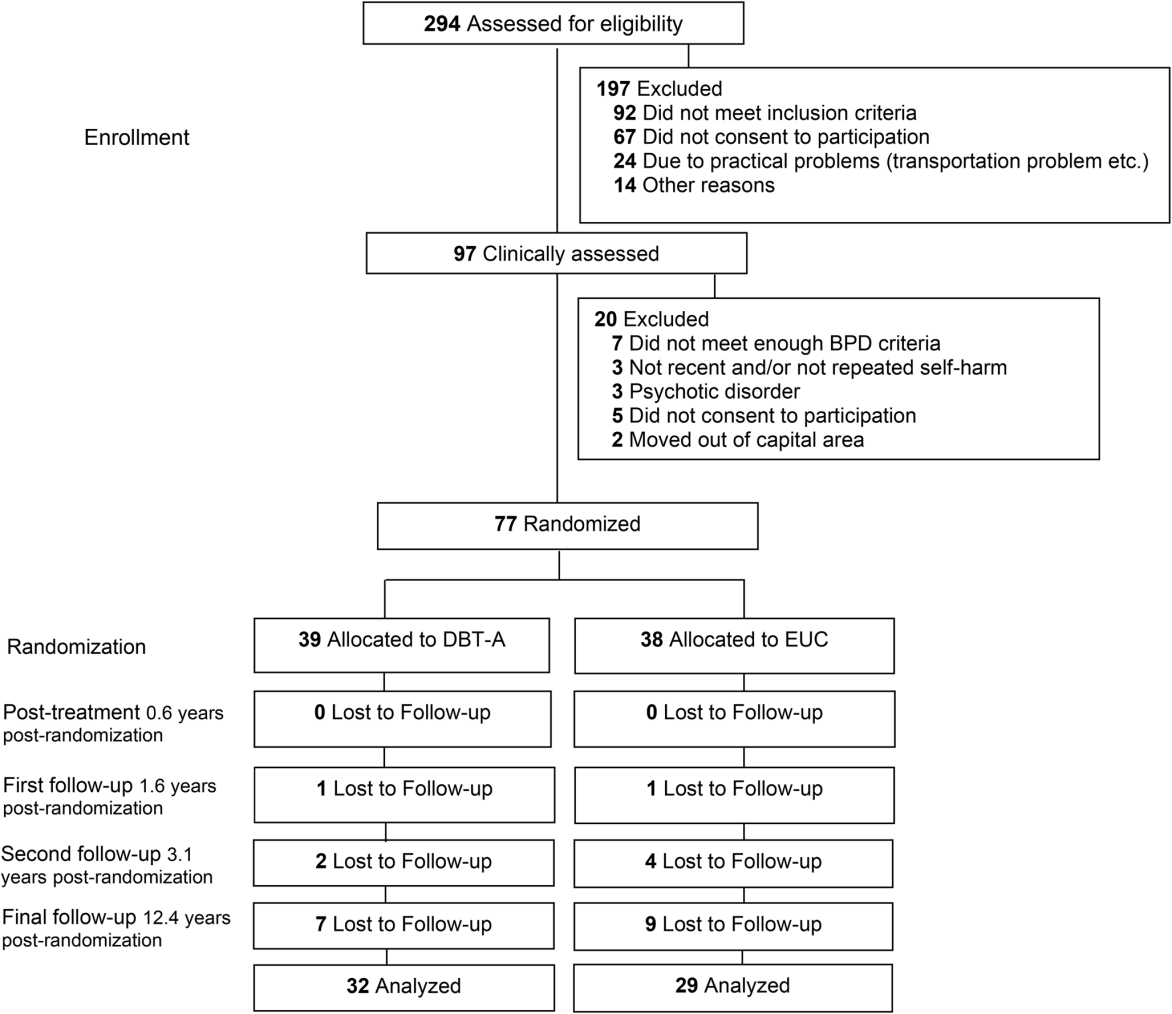
Flowchart (CONSORT) of participants in 12.4 years follow-up of a RCT comparing DBT-A and EUC for suicidal and self-harming behavior

Details on trial methods are published elsewhere [19] but, briefly, participants were included from child and adolescent psychiatric outpatient clinics in Oslo if they had a history of ≥ 2 episodes of self-harm, wherein ≥ 1 within the last 16 weeks, ≥ 3 criteria of DSM-IV BPD including the self-destructive criterion and Norwegian fluency. Exclusion criteria were bipolar I disorder, any psychotic disorder, intellectual disability or Asperger’s syndrome. The power analysis suggested that, with repetition of self-harm over a 19-week observation period as the primary outcome and an alpha error level of 0.05, a sample of 80 participants would be required to provide 80% power with a 2-tailed test. Eighty participants were indeed recruited, but three of these turned out to have a diagnosis of psychotic disorder soon after treatment start resulting in a final sample of 77 participants (12–18 years) enrolled and randomized to receive19 weeks of DBT-A or EUC (Fig. 1) in the period 2008 to 2012.

Allocation followed baseline assessments and was based on a permuted block randomization procedure with an undisclosed and variable blocking factor, and was performed by an external group. Participants were followed-up twice during the treatment, at end-of-treatment and then after 1.6 and 3.1 years; these outcomes have been reported previously [19, 22, 23]. An average of 12.4 years post-baseline all participants were invited, in the period 2021–2022, for a final follow-up from which key outcomes are reported in this paper. The study adhered to the principles of the Helsinki Declaration and was approved by the Regional Committee for Medical Research Ethics South East Norway. All participants and their legal guardians signed informed consent for participation.

### Treatments

DBT-A was delivered over 19 weeks, according to the manual developed by Miller, et al. [18] with treatment delivered within the adherent range [19]. EUC was 19 weeks of standard non-DBT treatment delivered at the participating child and adolescent outpatient psychiatric clinics, mainly psychodynamic or cognitive behavioral therapy. For all study participants psychopharmacological treatment could be received if needed [31].

### Measures

Baseline assessments, conducted prior to randomization, were made by two child and adolescent psychiatrists and two doctoral level clinicians. Further assessments occurred at 9-, 15- and 19-weeks post-baseline and 1.6-, 3.1- and 12.4-years post-baseline. Interview assessments at the 12.4-year assessment were made by a clinical psychologist blinded to treatment allocation.

#### *Primary outcome measures*

Frequency of suicide attempts (defined as self-harm with at least some suicide intent) and episodes of non-suicidal self-injury (NSSI) (defined as self-harm without any suicide intent) were reported through the *Linehan Parasuicide Count *[32] in all but the last follow-up where the *Suicide Attempt Self-Injury Interview* (*SASII) *[33] was used, since this interview enabled retrospective assessment of suicide attempts and NSSI episodes over such a long follow-up interval as in the present study.

#### *Secondary outcome measures*

*Suicidal ideation* was measured by the *Suicidal Ideation Questionnaire (SIQ-Jr) *[34]. Borderline symptoms by the *Borderline Symptom List-23 (BSL-23) *[35], and depressive symptoms clinician-rated with *Montgomery and Aasberg Depression Rating Scale (MADRS) *[36] and self-reported through the *Short Moods and Feelings Questionnaire (S-MFQ) *[37]*.* Hopelessness was measured by the *Beck Hopelessness Scale (BHS) *[38]*,* global level of functioning by the *Children's Global Assessment Scale (CGAS) *[39] for the first three timepoints, and through the *Global Assessment of Functioning (GAF)* scale [40] in the final two. Axis I diagnoses were made using the Mini International Neuropsychiatric interview (MINI) [41], and Borderline personality disorder diagnosed through the *SCID-II* for DSM-IV [42].

All follow-up interviews were audio-taped, and inter-rater reliability of diagnoses and interview rated outcome variables was checked by a psychologist expert in the relevant assessment instruments. Based on 24 randomly selected reliability-rated interviews, the mean Cohen's Kappa was 0.67 for axis I disorders. Weighted Cohen's Kappa for the BPD diagnosis was 0.76, whereas the mean Kappa for individual diagnostic criteria was 0.63. According to recommendations by McHugh [43] Cohen's Kappa values above 0.60 should be regarded as indicative of a moderate agreement between raters. Intra-class correlation (ICC) was used to test inter-rater reliability for GAF (GAF-S: ICC = 0.80, and GAF-F: ICC = 0.89), MADRS score (ICC = 0.95), and SASII (mean ICC = 0.98). According to Koo and Li [44] ICC values between 0.75 and 0.9 and greater than 0.90 are indicative of good and excellent reliability, respectively.

### Statistical analysis

Means with standard deviations or median with interquartile ranges were computed for normally and non-normally distributed variables, respectively. Differences between treatment groups were tested by independent sample t-tests and Pearson’s Chi-square tests. For the estimation of longitudinal effects of treatment, mixed linear models were used for the continuous outcome variables, with person-level random intercepts and sum scores as dependent variables. All time points were included, and time was treated as a categorical variable. Stratification variables used in the random allocation procedure (gender, presence of MDD and suicide attempt last 4 months) were included as covariates, and the Akaike information criterion was used in the model selection [45]. A generalized linear mixed effects model (GLMM) with negative binomial distribution was used to test for differences between the groups across time in frequency of self-harm episodes. The parametrization used to handle overdispersion was conditional mean. In all of the mixed effects models, treatment condition was included as a main effect and an interaction effect with time. Statistical significance was defined as a P-value less than 0.05. All statistical analyses were conducted in STATA 17.

## Results

Participation rate has been consistently very high in this study with 100%, 98% and 92% participation at end-of-treatment, 1.6 and 3.1-year follow-ups respectively. At the final 12.4-year follow-up 79% (*N* = 61) of the original sample participated (DBT-A, *n* = 32, EUC, *n* = 29) (Fig. 1). At this follow-up, participants were a mean of 28.7 years (SD = 1.9) and predominantly female (87%). There were no suicides, nor deaths of other causes. Sample characteristics are presented in Table 1.

**Table 1:** Sample characteristics, diagnostic data and self-harm outcomes at 12.4 years follow-up

Variable	DBT-A (*n* = 32)	EUC (*n* = 29)	Total (*N* = 61)	Statistic	*P* Value
Age, mean (SD)	28.48 (1.84)	27.61 (1.96)	28.07 (1.93)	t = 1.77	0.08
Female, No (%)	28 (88)	25 (86)	53 (87)	${\upchi }^{2}$= 0.04	0.83
Norwegian ethnicity, No (%)	28 (88)	25 (86)	53 (87)	${\upchi }^{2}$= 0.02	0.88
Education level, No (%)				${\upchi }^{2}=$ 3.46	0.48
Secondary education	3 (9)	6 (21)	9 (15)		
High school	11 (34)	10 (35)	21 (34)		
Some higher education	2 (6)	0	2 (3)		
Undergraduate	11 (34)	10 (35)	21 (34)		
Graduate	5 (16)	3 (10)	8 (13)		
Occupational status, No (%)				${\upchi }^{2}=$ 2.12	0.71
Full-time employed or student	18 (56)	20 (69)	38 (62)		
Part-time employed or student	7 (22)	3 (10)	10 (16)		
Unemployed	2 (6)	1 (3)	3 (5)		
Disabled > 50%	4 (13)	4 (14)	8 (13)		
On maternity/paternity leave	2 (6)	1 (3)	3 (5)		
Marital status, No (%)				${\upchi }^{2}=$ 5.12	0.28
Single	4 (13)	9 (31)	13 (21)		
Partner (not living together)	6 (19)	6 (21)	12 (20)		
Married/living with partner	21 (66)	14 (48)	35 (57)		
Divorced	1 (3)	0	1 (2)		
Current psychiatric disorders, No (%)					
Any mood disorder	8 (25)	12 (41)	20 (33)	${\upchi }^{2}=$ 1.85	0.17
Major Depressive Disorder	4 (13)	5 (17)	9 (15)	-	0.7
Dysthymia	3 (9)	3 (10)	6 (10)	-	1.00
Bipolar II disorder	1 (3)	4 (14)	5 (8)	-	0.18
Any anxiety disorder	14 (44)	11 (38)	25 (41)	${\upchi }^{2}=$ 0.21	0.64
Panic disorder/agoraphobia	9 (28)	6 (21)	15 (25)	${\upchi }^{2}=$0.45	0.50
Social phobia	7 (22)	7 (24)	14 (23)	${\upchi }^{2}=$0.04	0.83
Obsessive Compulsive Disorder	2 (6)	0	2 (3)	-	0.49
Generalized Anxiety Disorder	3 (9)	2 (7)	5 (8)	-	1.00
Any stress-related disorder	7 (22)	8 (28)	15 (25)	${\upchi }^{2}=$0.26	0.60
Post-Traumatic Stress Disorder	6 (19)	7 (24)	13 (21)	${\upchi }^{2}=$0.26	0.60
Acute stress disorder	1 (3)	1 (3)	2 (3)	-	1.00
Any substance use disorder	4 (13)	6 (21)	10 (16)	-	0.49
Alcohol misuse disorder	1 (3)	1 (3)	2 (3)	-	1.00
Alcohol dependence	2 (6)	4 (14)	6 (10)	-	0.41
Substance misuse disorder	1 (3)	2 (7)	3 (5)	-	0.60
Substance dependence	1 (3)	1 (3)	2 (3)	-	1.00
Schizophrenia	2 (6)	0	2 (3)	-	0.49
Any eating disorder	1 (3)	4 (14)	5 (8)	-	0.18
Bulimia nervosa	0	2 (7)	2 (3)	-	0.22
Eating disorder NOS	1 (3)	2 (7)	3 (5)	-	0.60
ADHD	4 (13)	4 (14)	8 (13)	-	1.00
Borderline Personality Disorder	3 (9)	5 (17)	8 (13)	-	0.46
Number of BPD criteria fulfilled, mean (SD)	1.66 (2.13)	1.97 (2.06)	1.8 (2.09)	*t* = 0.57	0.56
Number of current DSM-IV axis I diagnoses, mean (SD)	1.53 (1.48)	1.72 (1.65)	1.62 (1.55)	*t* = 0.47	0.63
No current psychiatric disorder	9 (28)	9 (31)	18 (30)	.${\upchi }^{2}=$ 06	0.80
Self-harm					
Any suicide attempt last year, No^1^ (%)	1 (3)	0 (0)	1 (2)	-	1.00
Any suicide attempt last 9.3 years, No^1^ (%)	6 (19)	5 (17)	11 (18)	${\upchi }^{2}=$0.02	0.87
Number of suicide attempts last year (median)	0	0	0	-	-
Any NSSI last year, No^1^ (%)	6 (19)	6 (21)	12 (20)	${\upchi }^{2}=$0.03	0.84
Total frequency of NSSI in group last year, No	28	8	36	-	-
Any NSSI last 9.3 years, No^1^ (%)	24 (75)	16 (55)	40 (66)	${\upchi }^{2}=$ 3.71	0.054
Total frequency of NSSI in group last 9.3 years, No	792	1727	2519	-	-
Mean frequency of NSSI episodes (SD)	24.75 (67.3)	59.55 (154.2)	41.83 (117.67)	*t* = −1.12	0.26
Symptom and function scores					
MADRS (score ranges), mean (SD)	11.72 (8.76)	13.55 (9.21)	12.59 (8.96)	*t* = −0.79	0.42
No depression (0–6), No (%)	11 (34)	8 (28)	19 (31)	${\upchi }^{2}=$0.32	0.56
Mild depression (7–19), No (%)	14 (44)	14 (48)	28 (46)	${\upchi }^{2}=$0.12	0.72
Moderate depression (20–34), No (%)	7 (22)	6 (21)	13 (21)	${\upchi }^{2}=$0.01	0.91
Severe depression (> 34), No (%)	0 (0)	1 (4)	1 (2)	-	0.47
SMFQ, mean (SD)	9.00 (6.58)	9.27 (7.29)	9.13 (6.88)	${\upchi }^{2}=$−0.15	0.87
Suicidal Ideation (SIQ-JR), mean (SD)	18.46 (14.10)	24.97 (21.36)	21.78 (18.29)	${\upchi }^{2}=$−1.36	0.18
Hopelessness (BHS), mean (SD)	5.33 (5.62)	6.51 (5.67)	6.00 (5.62)	${\upchi }^{2}=$−0.72	0.47
Borderline symptoms (BSL-23), mean (SD)	18.48 (13.16)	23.65 (21.27)	20.98 (17.59)	${\upchi }^{2}=$−1.12	0.26
Alcohol use (AUDIT-C), mean (SD)	3.94 (2.09)	4.62 (2.51)	4.26 (2.30)	${\upchi }^{2}=$−1.14	0.25
GAF-S, mean (SD)	65.69 (12.58)	65.62 (14.70)	65.65 (13.50)	t = 0.01	0.98
GAF-F, mean (SD)	66.87 (13.45)	68.10 (15.64)	67.46 (14.41)	t = −0.33	0.74

^1^Number of participants with any episode of the behaviors over the relevant time interval

Note: For categorical variables with < 5 observations, Fisher’s exact test was calculated

*DBT-A* Dialectical Behavior Therapy adapted for Adolescents, *EUC* Enhanced Usual Care, *ADHD* Attention Deficit Hyperactivity Disorder, *BPD* Borderline Personality Disorder, *NSSI* Non-Suicidal Self-Injury, *MADRS* Montgomery and Asberg Depression Rating Scale, *SMFQ* Short Mood and Feelings Questionnaire, *SIQ-Jr* Suicidal Ideation Questionnaire - Junior, *BHS* Beck Hopelessness Scale, *BSL-23* Borderline Symptom List - 23 items version, *AUDIT-C* Alcohol Use Disorders Identification Test Consumption, *GAF-S* Global Assessment of Functioning - Symptom level, *GAF-F* Global Assessment of Functioning - Function level

### Primary outcomes

#### *Self-harm episodes*

**Fig. 2:**
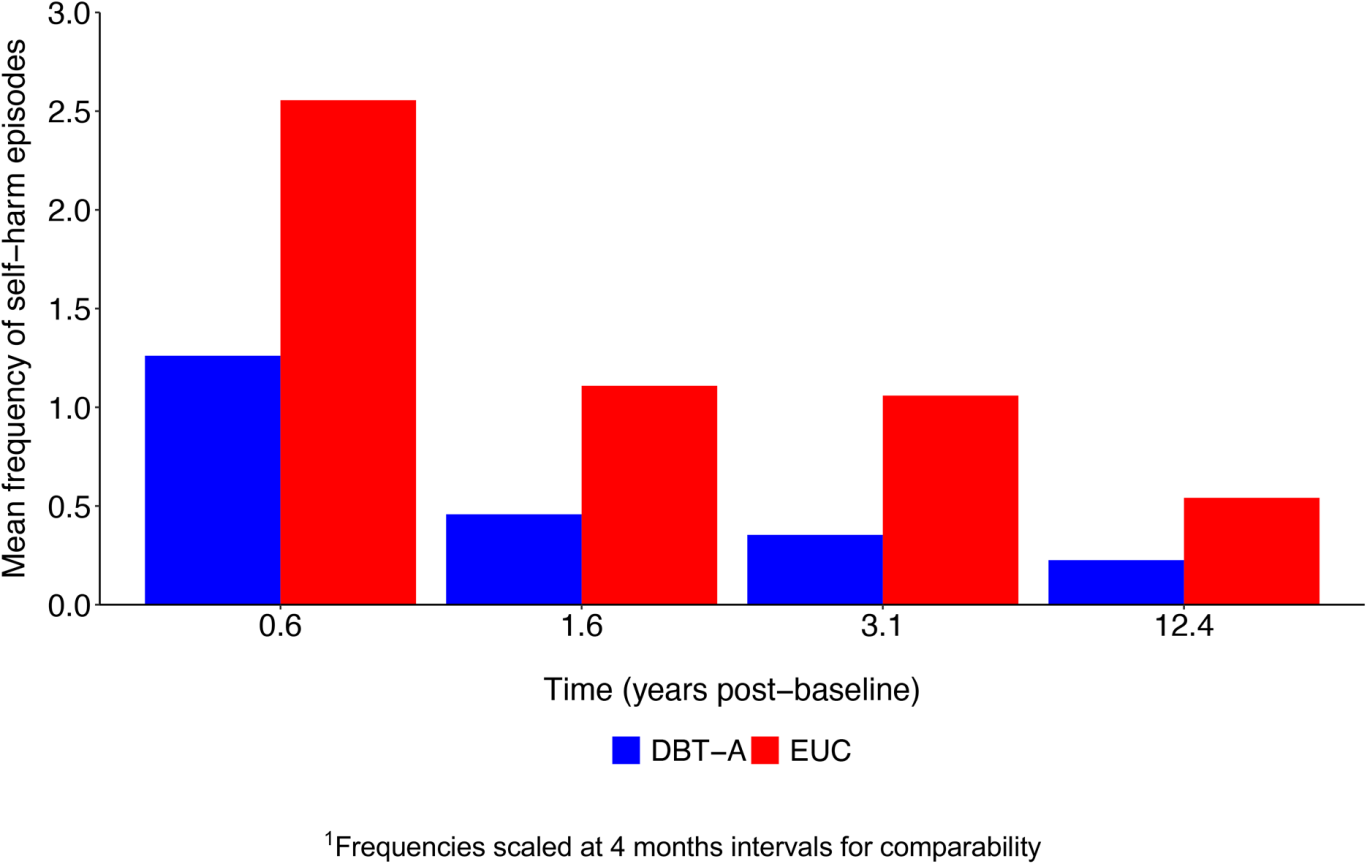
Frequency of Self-harm Episodes over 12.4 years post-baseline^1^ in Participants of a RCT of DBT-A vs. EUC for Suicidal and Self-harming Behavior. DBT-A - Dialectical Behavior Therapy adapted for Adolescents, EUC - Enhanced Usual Care

The mean total frequency of self-harm episodes (encompassing suicide attempts and NSSI) in the period between the 3.1- and 12.4-year follow-ups was 25.1 (SD = 67.3) in the DBT-A group and 60.3 (SD = 154.9) in the EUC group. How self-harm frequency changed over the entire 12.4-year interval among participants in the two treatment conditions is shown in Fig. 2 (frequencies scaled at 4-month intervals for comparability between unequal time intervals).

GLMM analyses with negative binomial distribution to account for the over-dispersion and left-skewness of self-harm episodes showed that incident rate ratios (IRR) for self-harm were consistently higher in the EUC group compared to the DBT-A group at all follow-up intervals (Table 2), not reaching statistical significance for the last interval with few episodes in both groups.

**Table 2:** Incidence Rate Ratios (IRR) for frequency of self-harm scaled at 4 follow-up intervals among study participants

Years post-baseline	IRR^1^	SE	*p*-value	95% CI
0.0 - 0.6	3.44	1.82	0.02	1.22–9.72
0.6 - 1.6	7.01	3.89	<.001	2.36–20.78
1.6 - 3.1	4.46	2.40	0.005	1.55–12.83
3.1 - 12.4	1.35	0.66	0.53	0.52–5.53

^1^IRR’s are presented for the time*treatment interaction term with DBT-A as the reference. Analyses were adjusted for stratification variables (gender, presence of major depression, and presence of suicide intent during the most serious episode of self-harm behavior within the 16 weeks before enrolment)

At every assessment interval, EUC was associated with increased incidence rate ratio of self-harm episodes relative to DBT-A, with the largest difference at the 1.6-year follow-up. Since, at the final follow-up interval, the proportion of participants reporting self-harm was low, a corresponding Poisson model was run as a reference to compare model fit, however the Akaike criteria clearly favored the negative binomial model. The area under a piecewise linear curve between the DBT-A and EUC groups corresponded to 5 869 self-harm episodes, or a mean difference of 90 episodes per person from baseline to 12.4 years follow-up.

#### *Suicide attempts*

Six participants in the DBT-A group reported any suicide attempts in the period between the 3.1 and 12.4-year follow-up (within-group range 1–4, median: 1.5), compared to five in the EUC group (within-group range 1–7, median: 5) (Table 1). One participant from the DBT-A group reported to have made a suicide attempt over the last year before the 12.4-year follow-up, and none in the EUC group.

#### *Non-suicidal self-injury*

During the last year 80% of participants (81% and 79% in DBT-A and EUC, respectively) reported to have had no NSSI; the mean total frequency in both groups was less than 1. Six participants in each group reported any NSSI during the last year, with a range of 1–20 episodes (1 participant in the DBT-A group reported 20 episodes). Over the period from 3.1 to 12.4-year follow-up a mean total NSSI frequency of 24.8 (SD = 67.3) was reported in the DBT-A group and 59.5 (SD = 154.2) in the EUC group. The majority of participants who reported NSSI during this interval had between 1 and 9 episodes, on average less than one episode per year. Examining potential treatment group differences among participants reporting more than 10 episodes (> the 75th percentile of the frequency distribution) we conducted a quantile regression analysis showing no significant difference between treatment conditions. However, a difference was apparent at the 90^th^ percentile (β = 231, SE: 96.7, *p* = 0.018), such that EUC participants were significantly more likely to report NSSI frequencies above this percentile than DBT-A participants.

### Secondary outcomes

#### *Suicidal ideation, borderline and depressive symptoms*

**Fig. 3:**
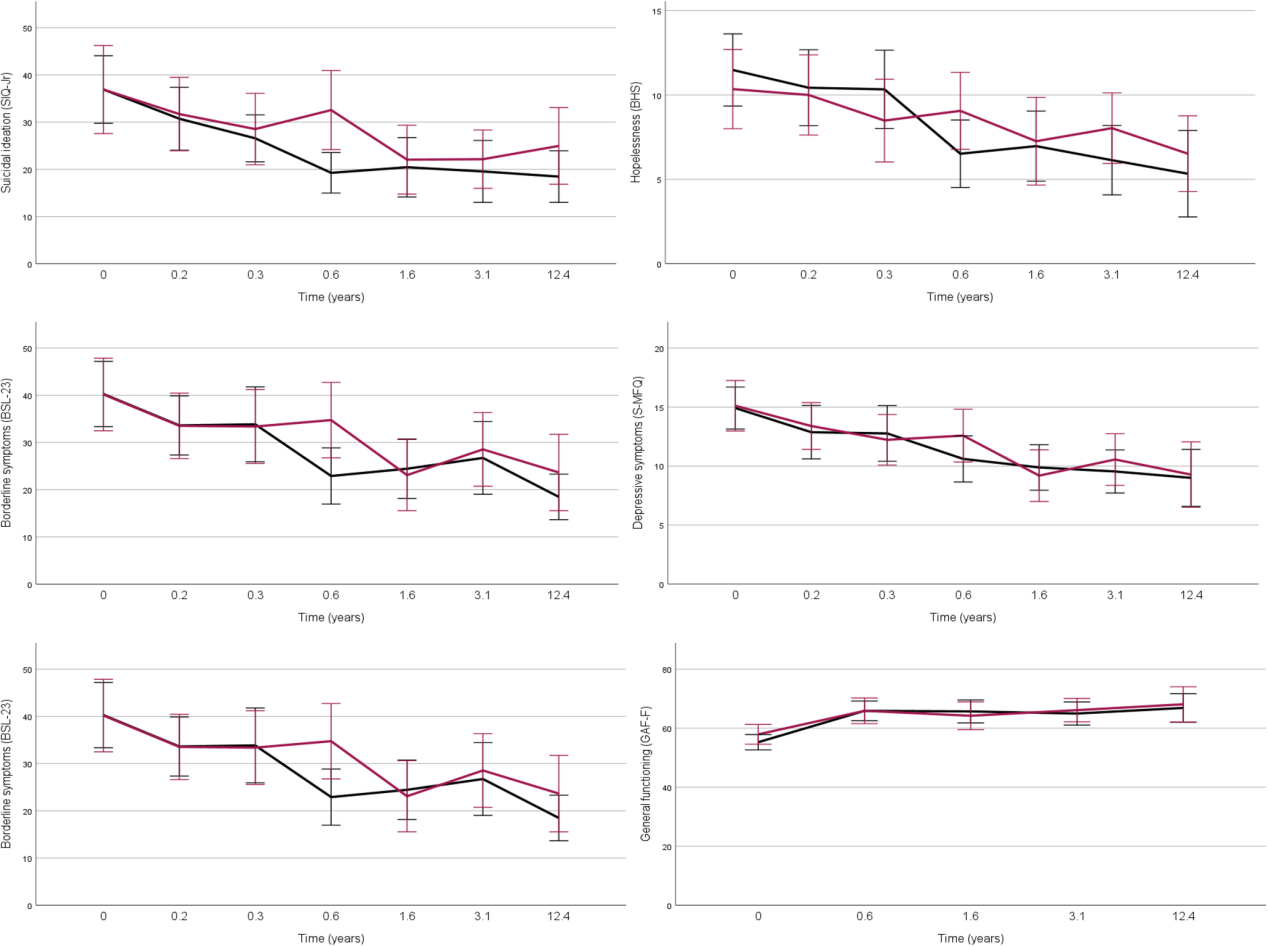
Outcomes over 12.4 years Follow-up in Participants of a RCT of DBT-A vs. EUC for Suicidal and Self-harming Behavior. DBT-A - Dialectical Behavior Therapy adapted for Adolescents, EUC - Enhanced Usual Care, MADRS - Montgomery and Asberg Depression Rating Scale, SMFQ - Short Mood and Feelings Questionnaire, SIQ-Jr - Suicidal Ideation Questionnaire - Junior, BHS - Beck Hopelessness Scale, BSL-23 - Borderline Symptom List - 23 items version, CGAS - Children's Global Assessment Scale, GAF - Global Assessment of Functioning

We found decreases in all symptom scores with time in both treatment groups, but no between-group differences were observed in suicidal ideation, borderline symptoms and clinician-rated depressive symptoms (Fig. 3).

Linear mixed models with random intercepts and time as a categorical variable, with and without baseline adjustment of the variables of interest, demonstrated significant time*treatment interactions for changes in depressive symptoms (MADRS) (β = 1.02, SE = 0.43, *p* = 0.020), borderline symptoms (BSL-23) (β = 1.01, SE = 0.49, *p* = 0.041) and suicidal ideation (SIQ-Jr) (β = 2.03, SE = 0.79, *p* = 0.011) all in favor of DBT-A at 0.6 years post-treatment. In all models, baseline adjustment reduced the estimates slightly, and the treatment*time effect on borderline symptoms was reduced, while the MADRS and SIQ-Jr effects remained nearly unchanged (Table 3). There were no significant differences in global functioning (C-GAS/GAF) or self-rated depressive symptoms (S-MFQ). For clinician-rated depressive symptoms (MADRS) relatively few participants reported symptoms within the moderate to severe range (7 in each treatment condition), the majority reported in the mild depression range (Table 1).

**Table 3:** Regression coefficients for the effect of time*treatment condition on suicidal ideation, borderline symptoms and depressive symptoms

Variables	Coef. β time*treatment	*p*-value	Coef. β time*treatment	*p*-value
	Unadjusted		Baseline adjusted	
SIQ-Jr	1.49	0.023	1.60	0.012
BSL-23	0.99	0.012	0.91	0.027
MADRS	0.80	0.027	0.91	0.012

Only estimates for T4 (post treatment) are shown. All estimates were adjusted for stratification variables (sex, presence of major depressive disorder and suicide attempt over the past 4 months at baseline)

SIQ-Jr - Suicidal Ideation Questionnaire Junior, BSL-23 - Borderline Symptom List 23 items version, MADRS - Montgomery and Asberg Depression Rating Scale

#### *Borderline personality disorder diagnosis*

Eight participants (DBT-A: 3, EUC: 5) met criteria for a full BPD diagnosis at the 12.4-year follow-up. In the DBT-A group, BPD was reduced from 28 to 9%, while it increased from 15 to 17% in the EUC group, these differences were not statistically significant. Whereas all participants met the recurrent self-harm criterion for BPD at baseline, only six participants did so at the 12.4-year follow-up; two in the DBT-A group and four in the EUC group.

#### *Psychosocial and occupational functioning*

Half of the participants had completed at least some higher education, while 15% had finished only secondary education; 9% in the DBT-A group and 21% in the EUC group. Overall, the majority of participants were at least part-time employed, whereas 18% were unemployed or receiving disability compensation (Table 1). Among participants with absence from work the past four weeks, mean number of days absent were 3.9 (SD = 3.8) in the DBT-A group and 8.2 (SD = 9.9) in the EUC group. A third of the participants in the EUC group were single, compared to 13% in the DBT-A group. Altogether 19% of participants in the DBT-A group had children, compared to 10% in the EUC group. Overall, GAF scores had slightly increased since the prior follow-up, with mean scores of 65.65 (SD = 13.50) for symptoms and 67.46 (SD = 14.41) for functioning (Table 1). A majority of participants in both groups scored above the cut-off (61) (74% in the DBT-A group and 79% in the EUC group). None of the between-group differences in psychosocial functioning were statistically significant.

## Discussion

To our knowledge, this is the first randomized study of the long-term effectiveness of any psychosocial treatment for adolescents spanning the time from study inclusion during mid-adolescence into adult life. Self-harm frequency was consistently lower throughout this long follow-up period in participants who had received DBT-A compared to EUC, implying a mean difference of 90 self-harm episodes. There were relatively few suicide attempts over the post-treatment follow-up intervals and nearly none during the last follow-up year. NSSI episodes were more than twice as common in the EUC group as the DBT-A group. Still, 80% of participants reported no NSSI during the last follow-up year. DBT-A was associated with significantly stronger reductions in suicidal ideation, borderline symptoms and clinician-rated depressive symptoms at end of treatment, whereas there were no significant long-term trend differences in these outcomes or in current psychiatric morbidity, self-reported depressive symptoms and global functioning.

### Suicidal and non-suicidal self-harm behavior

Rates of self-harm in adolescents naturally decrease into adulthood shown in studies from the general population [13, 46]. Whether there is a similar trend in clinical samples is unclear. That nearly 80% of our EUC participants reported no last-year self-harm suggests that remission eventually occurs even without specialized treatment specifically targeting self-harm. The big difference between the treatment groups in this study is, however, the time at which remission occurred; on average several years earlier in DBT-A participants, as we have reported in a previous paper [47], with the consequences such an early remission of self-harm is likely to have in terms of improved safety, stability and quality of life at this important developmental stage. Recurrent self-harm behavior has been shown to increase the likelihood of subsequent suicide attempts [7, 9] and suicide death [8] in adolescents, thus, to implement effective interventions to reduce future self-harm would be a logical preventive strategy. As we have demonstrated in a previous paper from the current trial, subjects who had achieved self-harm remission within one year after treatment completion displayed significantly lower levels of emotion regulation problems as adults [47]. While the exact mechanisms through which self-harm remission and emotion regulation capacity may be interconnected are still unknown, it seems likely that self-harm as a coping strategy may prevent adolescents from developing more healthy coping skills.

Interestingly, although the mean frequency of self-harm over the preceding 9.3 years was lower in the DBT-A group, a higher number of participants reported having engaged in any NSSI. This might be explained by differences in attrition between the treatment groups, i.e. that more DBT-A participants participated in the study over the long-term follow-up interval. However, it is also possible that there were real differences between the groups in their patterns of self-harm behavior. Hawton et al., [48] have argued in favour of distinguishing sporadic episodes of self-harm associated with acute crises or distress from more recurrent and habitual patterns associated with emotion regulation problems. Young adults with a pattern of sporadic NSSI have been found to be more similar to peers reporting NSSI cessation, compared to those with patterns of persistent NSSI [49]. Indeed, in a previous study we did find that lack of self-harm remission one year after treatment completion was more strongly associated with emotion dysregulation in adulthood EUC participants [47].

### Borderline pathology

Previous studies have indicated the ages around 14–17 as a critical period where symptoms of BPD peak [29]. It would be logical to deliver interventions during periods of highest risk, and to assume that intervening at this critical time could be most effective to positively influence the trajectory of BPD. DBT-A was indeed associated with statistically significant larger reductions in BPD symptoms after treatment in our study, and the proportion meeting criteria for a BPD diagnosis dropped from 28 to 9%, whereas no such change was observed in the EUC group. This diagnostic difference was not statistically significant and would need to be re-examined in a larger sample. Our finding, nevertheless, contrasts with previous studies [28] where no changes have been found in the amount of BPD pathology over a longer follow-up after treatment. Inclusion in our trial required fulfilment of at least 3 BPD diagnostic criteria, not a full diagnosis since adolescents with recurrent self-harm who meet diagnostic criteria for a full-syndrome BPD or sub-threshold BPD seem to have difficulties within the same spectrum [24]. They seem dimensionally, but not categorically, different regarding the severity of their personality difficulties. To provide interventions to a broader clinical group of adolescents with borderline features targeting their dysfunctional self-harm behavior and BPD symptoms at an earlier, rather than at a later stage of symptom development would seem important, although more studies with larger samples are needed to clarify this.

### Clinical implications

In previous papers from this trial [19, 47] we have demonstrated that adolescents who received DBT-A experienced significantly earlier improvement in frequency of self-harm episodes, suicidal ideation and borderline pathology. This suggests that, if given effective treatments, such as DBT-A, adolescents may have a realistic hope of overcoming recurrent self-harm, suicidality and borderline pathology at a faster rate than if they had received usual care. In a separate paper from the same study, we have also demonstrated the importance of attaining an early remission of self-harm behavior for developing a capacity to regulate emotions in healthy ways in adult life [47]. Thus, treatment programs for adolescents with these problems should actively and effectively target self-harm and emotion dysregulation[50]. At the same time, clinicians should be aware that a strong focus on self-harm reduction may not be what adolescents wish for, since, after all, this behavior serves important emotion regulation functions [51]. Treatment programs need also to take adolescents' values and needs into account when treatment targets are outlined; these targets should always embrace general functioning, health and quality of life [17]. Early detection of recurrent self-harm behavior is of high importance, since the younger age at which it starts and the longer it is allowed to last without any treatment, the higher the frequency of self-harm episodes and the higher the likelihood of eventual suicide attempts [7].

### Limitations and strengths

Among study strengths are the prospective design, very long follow-up period, the high integrity of ratings, blinding and independence of raters and the nearly 80% participation rate even after 12.4 years. Among limitations are the relatively small sample size and, although the study was adequately powered, findings should, thus, be interpreted with caution. Another limitation is retrospective reporting of NSSI episodes over such a long follow-up period, although this is not likely to influence between-group differences. As in most trials of self-harm, the sample was mostly female and too small to study gender differences in long-term treatment outcomes.

## Conclusion

This adult life follow-up of a randomized trial of DBT-A vs EUC for adolescents with recurrent suicidal and self-harming behavior and borderline features showed that DBT-A was associated with consistently lower self-harm rates throughout the 12.4 year follow-up.

## Data Availability

The terms under which participants signed informed consent the study does not allow for making data publicly available.

## Footnotes

1 Our study was pre-registered as a 10-year post-baseline follow-up, but was postponed more than 2 years due to the Covid-19 pandemic.
